# Vector competence of European mosquitoes for West Nile virus

**DOI:** 10.1038/emi.2017.82

**Published:** 2017-11-08

**Authors:** Chantal BF Vogels, Giel P Göertz, Gorben P Pijlman, Constantianus JM Koenraadt

**Affiliations:** 1 Laboratory of Entomology, Wageningen University & Research, P.O. Box 16, 6700 AA, Wageningen, The Netherlands; 2 Laboratory of Virology, Wageningen University & Research, P.O. Box 16, 6700 AA, Wageningen, The Netherlands

**Keywords:** Europe, innate immune responses, midgut barrier, mosquito, salivary gland barrier, surveillance, vector competence, West Nile virus

## Abstract

West Nile virus (WNV) is an arthropod-borne flavivirus of high medical and veterinary importance. The main vectors for WNV are mosquito species of the *Culex* genus that transmit WNV among birds, and occasionally to humans and horses, which are ‘dead-end’ hosts. Recently, several studies have been published that aimed to identify the mosquito species that serve as vectors for WNV in Europe. These studies provide insight in factors that can influence vector competence of European mosquito species for WNV. Here, we review the current knowledge on vector competence of European mosquitoes for WNV, and the molecular knowledge on physical barriers, anti-viral pathways and microbes that influence vector competence based on studies with other flaviviruses. By comparing the 12 available WNV vector competence studies with European mosquitoes we evaluate the effect of factors such as temperature, mosquito origin and mosquito biotype on vector competence. In addition, we propose a standardised methodology to allow for comparative studies across Europe. Finally, we identify knowledge gaps regarding vector competence that, once addressed, will provide important insights into WNV transmission and ultimately contribute to effective strategies to control WNV.

## WEST NILE VIRUS IN EUROPE

**Figure 1:**
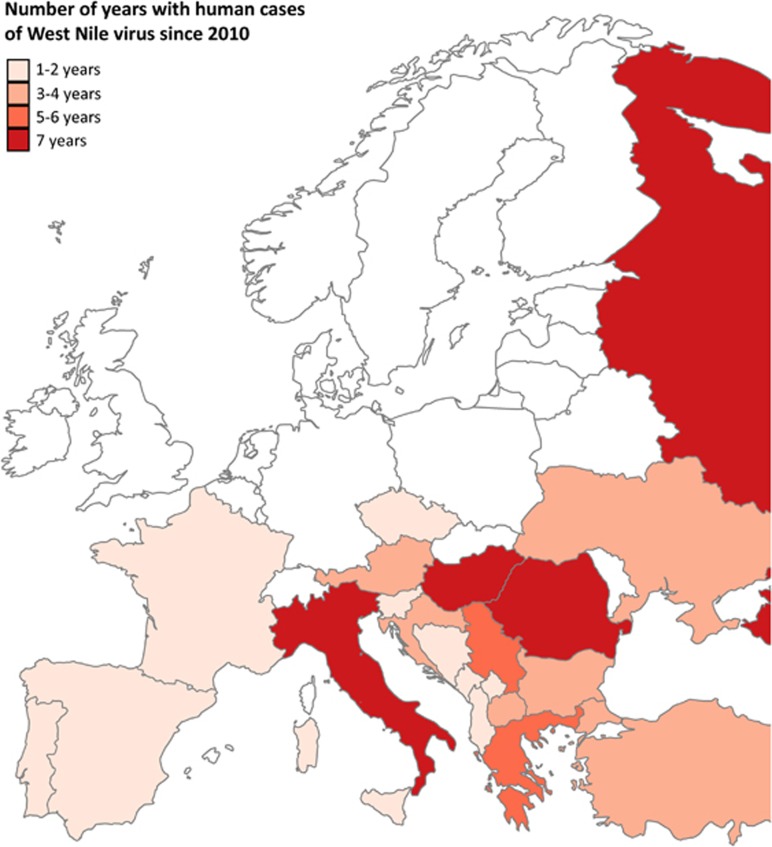
European countries with human cases of West Nile fever reported to the European centre for disease prevention and control (ECDC). Colour gradient indicates the number of years during which cases of West Nile virus in humans have been reported since 2010. Reported cases are marked on the country level, except for the Balearic islands, Sicily, Sardinia, and Corsica, which are individually marked. Countries with no reported cases or no data available are marked in white. Data set provided by ECDC based on the data provided by WHO and Ministries of Health from the affected countries.

Natural transmission cycles of the arthropod-borne West Nile virus (WNV; family: *Flaviviridae*) can only be established if susceptible bird hosts and competent mosquito vectors are present in a certain area under suitable environmental conditions. Mammals, including humans and equines, can occasionally become infected with WNV, but they generally do not develop sufficient viraemia to sustain transmission (reviewed in Bowen and Nemeth^1^). Thus, mammals are considered as ‘dead-end’ hosts. Most WNV infections in humans remain asymptomatic, although an estimated one in four cases develops into West Nile fever (reviewed in Petersen *et al.*^2^). Less than one percent of human WNV infections results in neurological disease such as meningitis or encephalitis, which can be fatal. Thus far, cases of WNV infection in humans have only been reported from southern and central European countries (Figure 1). Due to absence of human and equine cases in northern European countries, monitoring of WNV in these regions mainly depends on mosquito and bird surveillance programmes. Since routine surveillance programmes are not running in all European countries, and as most WNV infections in humans are asymptomatic, WNV circulation is likely to be underestimated.

The differences in the number of reported WNV cases in humans between northern and southern Europe may be explained by two main hypotheses: (i) lower susceptibility of northern European bird populations and (ii) lower vectorial capacity of northern European mosquito species. Studies from Germany and the United Kingdom have shown serological evidence of WNV circulation among resident birds.^3, 4, 5^ In addition, laboratory studies with carrion crows (*Corvus corone*),^6^ and European jackdaws (*Corvus monedula*),^7^ showed their susceptibility to infection with WNV. Viraemia of carrion crows (50 % tissue culture infectious dose: >10^5^ TCID_50_/mL) was found to be sufficient to support WNV transmission, but this was not the case for most of the jackdaws (⩽10^5^ TCID_50_/mL).^6, 7^ The combined evidence of both field and laboratory studies on northern European birds, indicates that bird hosts are likely not a limiting factor for WNV transmission in northern Europe. Although corvid birds are highly susceptible to WNV infection, studies in North America have shown that other species such as the American robin (*Turdus migratorius*), contribute proportionally more to the WNV transmission cycle.^8, 9, 10^ The larger contribution of American robins is mainly due to the high feeding preference of the main WNV mosquito vectors for this bird species. Although several studies determined feeding preferences of European mosquitoes,^11, 12, 13, 14, 15^ in-depth studies on spatial and temporal host-feeding dynamics of specific mosquito species remain scarce. A recent study on *Culex* (*Cx.*) *pipiens* mosquitoes originating from Italy showed that blackbirds (*Turdus merula*) and Eurasian magpies (*Pica pica*) were highly preferred by these mosquitoes.^14^ More studies focussing on the identification of host-feeding preferences of mosquitoes are needed to make a thorough comparison between mosquito–bird contact rates across a larger European scale. Such a comparison will provide important insights in key mosquito vector species and key bird host species, which should be tested for their susceptibility to WNV.

Lowered vectorial capacity of northern European mosquitoes can be an alternative explanation for the differences in WNV outbreaks across Europe. Vectorial capacity can be defined as the efficiency of a vector to transmit a pathogen.^16^ Vectorial capacity relies on several factors, such as the abovementioned feeding behaviour of mosquitoes, mosquito abundance, mosquito survival and environmental conditions. One of the key components of vectorial capacity is vector competence, which is defined as the ability of a vector to acquire, maintain and transmit a pathogen.^17^ Only competent vectors can contribute to the transmission of WNV, which makes vector competence an important determinant of vectorial capacity. However, a highly competent mosquito species may not efficiently contribute to WNV transmission if it only feeds on mammals and not on birds. Nevertheless, vector competence provides important insights in species that should be considered as important contributors to WNV transmission. This is necessary for targeted control of highly competent mosquitoes to reduce the potential of WNV transmission. In this review we aim to provide a complete and detailed overview of factors that influence vector competence of mosquitoes in Europe.

Thus far, most vector competence studies on WNV have been carried out with North-American mosquito species.^18, 19, 20, 21^ However, differences in vector species and environment make it difficult to extrapolate results from studies with North-American mosquitoes to understand the European situation. Until 2014, only two published studies had evaluated the vector competence of *Culex* species originating from Europe for WNV.^22, 23^ Although these studies provided insight into the possibility of WNV transmission by European mosquito species, they could not explain why WNV outbreaks seem limited to southern and central Europe. As the European Commission was concerned about further spread of WNV across Europe, several studies were initiated to investigate vector competence of European mosquitoes for WNV. As a result, several new vector competence studies on European mosquito species have been published during the past 3 years.

This review shows the results of vector competence studies on European mosquito species in order to identify key factors that influence vector competence for WNV. The concept of vector competence is explained, including the barriers to arbovirus infection of mosquitoes. Studies on vector competence of European mosquito species for WNV are being evaluated based on their methodology and outcomes to provide recommendations for future vector competence studies. The outcomes of the vector competence studies are linked to the available literature on mosquito barriers, immune pathways and interactions with other microbes that together determine vector competence. Finally, recommendations for WNV surveillance in Europe and perspectives for future research are discussed.

## MOSQUITO BARRIERS TO ARBOVIRUS INFECTION AND TRANSMISSION

**Figure 2:**
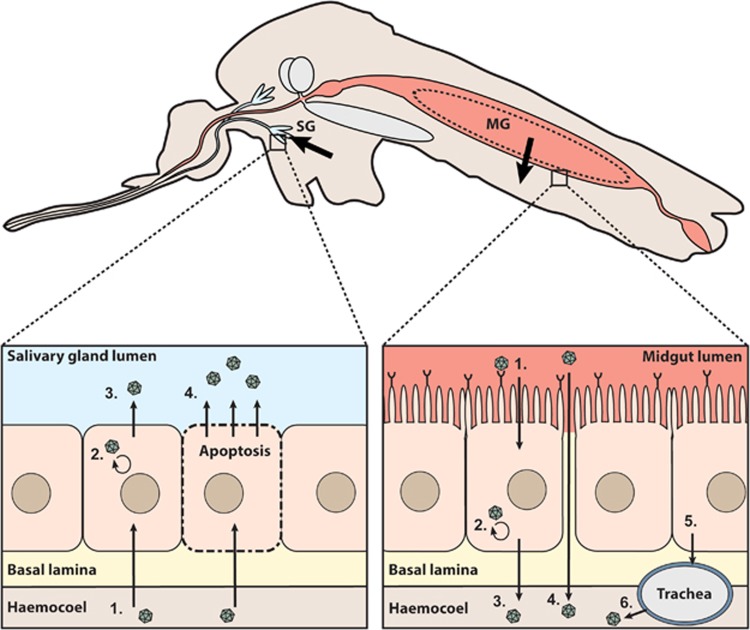
Schematic overview of the mosquito barriers to arbovirus infection. Schematic longitudinal cross-section of a mosquito. Arrows indicate the passage of virions through the midgut (MG) and salivary gland (SG) barriers. The dashed circle in the midgut represents the peritrophic membrane that is formed after ingestion of blood. Right inset: (i) Infection of midgut epithelial cells via binding to a putative receptor protein. (ii) Virus replication in midgut epithelial cells. (iii) Release of virus via budding from midgut epithelial cells and direct passage through the basal lamina into the haemocoel. (iv) Direct virus passage into the haemocoel through a ‘leaky’ midgut. (v) Virus infection of trachea after budding from midgut epithelial cells. (vi) Budding of virus from the trachea into the haemocoel. Left inset: (i) Infection of the salivary gland epithelial cells after passage through the basal lamina. (ii) Virus replication in the salivary gland cells. (iii) Virus release via budding from salivary gland cells into the salivary gland lumen. (iv) Virus release from the salivary gland cells into the salivary gland lumen via apoptosis.

The outcome of the interaction between mosquito and WNV is largely dependent on the specific combination of the mosquito species, mosquito origin, WNV lineage and WNV strain. For a virus-exposed mosquito to become infectious, the virus has to overcome various barriers within the mosquito body: the peritrophic membrane, the midgut barrier and the salivary gland barrier. The midgut and salivary gland barriers are both further divided into an infection and an escape barrier (Figure 2).^17^ These barriers can limit virus infection both mechanically and through a range of antiviral immune responses, thereby determining the vector competence of the mosquito to transmit a certain arbovirus.

After ingestion of an infectious blood meal, virus particles travel through the foregut, cardia (proventriculus; foregut-midgut junction), and eventually end up in the midgut. Although infection of the foregut and cardia has been described for some arboviruses,^24^ the majority of virus infections occur in the midgut epithelial cells (Figure 2; right inset). The first potential mosquito barrier that arboviruses encounter is the peritrophic membrane. The peritrophic membrane is a sac-like structure composed of chitin, proteins and glycoproteins that form a filamentous matrix surrounding the blood meal in the midgut.^25^ The peritrophic membrane is not constantly present in adult mosquitoes, but forms within a few hours after uptake of a blood meal. In *Culex* species, formation of the peritrophic membrane can be readily observed at 2–8 h post blood feeding, and reaches its peak thickness of ~100 μm after 24 h.^26^ The ~20 nm pores of the peritrophic membrane,^27^ are smaller than the ~50 nm diameter of WNV virions.^28^ This makes it necessary for WNV to infect the midgut epithelial cells within 8 h after ingestion of the viraemic blood meal.^27, 28^ Indeed, flaviviruses can infect the midgut epithelial cells within 8 h after a blood meal, and knock-down of genes required for the formation of the peritrophic membrane does not affect flavivirus infection in mosquitoes.^29, 30^ Thus, despite the limited literature available, the peritrophic membrane does not seem to pose a strong barrier against WNV and other flaviviruses in mosquitoes.

Entry of flaviviruses into the midgut epithelial cells most likely occurs through interaction with cellular membrane-associated receptors (Figure 2; right inset). Flavivirus receptors in mosquitoes have not yet been identified, although some studies have suggested the involvement of cellular proteins for dengue virus (DENV) entry in C6/36 mosquito cells.^31, 32^ These proteins are required for the binding and uptake of virions during DENV infection and are either membrane-bound or membrane-associated intracellular proteins.^32^ Studies with WNV in *Culex* mosquitoes indicated that infection of the midgut can be observed by the formation of irregular virus positive clusters of midgut epithelial cells at 3 days post initial midgut infection (dpi).^33^ At 5 dpi the virus spreads more widely throughout the midgut and fully infects the midgut at 7 dpi. Studies with WNV virus-like-particles showed that initial infections occur randomly across the luminal posterior midgut epithelium, suggesting that most cells in the posterior midgut are susceptible for virus entry.^34^

After infection and replication in the midgut epithelial cells, virus particles need to disseminate through the haemocoel in order to reach the salivary glands. The haemocoel can be reached by passing through the basal lamina with or without a virus replication cycle in the midgut epithelial cells, through infection of the midgut tracheae or through direct budding into the haemolymph (Figure 2). The basal lamina is a dense matrix of proteins that blocks large entities such as bacteria, fungi, and larger viruses. However, the basal lamina is permeable to small molecules, potentially forming a barrier for flavivirus dissemination to the haemocoel.^35^ The thickness of the midgut basal lamina differs between different mosquito species and strains, and can affect the ability of virions to pass to the haemocoel. Differences in thickness of the basal lamina of *Aedes (Ae.) albopictus* did not affect the dissemination of DENV-1.^36^ In addition, WNV particles can be observed within the intact basal lamina which suggests that WNV virions move freely within the lamina.^28^ These observations suggest that the basal lamina does not pose a strong barrier to flaviviruses. An alternative way of reaching the haemolymph is via infection of the tracheae. These form a network of tubular structures that reach from the midgut epithelial cells to the outer layer of the basal lamina. This network can sustain virus passage through the basal lamina.^37^ Indeed, DENV-2 can infect the tracheal system and infection of the tracheae is positively correlated with virus dissemination.^38^ Furthermore, it has been shown that the midgut tracheae of *Cx. pipiens* can serve as a conduit for Rift Valley fever virus passage through the basal lamina.^39^ Finally, it has been shown that a small proportion of mosquitoes can have a ‘leaky’ midgut, which refers to the direct passage of virions into the haemolymph immediately after an infectious blood meal, without a replication cycle in the midgut epithelial cells.^26^, ^40^ Although leaky midguts have not been shown as a characteristic of flavivirus infections, it is quite possible that in a small proportion of mosquitoes an infectious blood meal leads to direct infection of the haemolymph. To summarize, while most WNV infections pass through the midgut epithelial cells, some might pass this barrier by infection of the tracheae or directly through the leaky midgut.

After infection of the haemolymph, virions disseminate towards and infect secondary tissues including the fat body, haemocytes, muscles and ultimately the salivary glands. After infection of the salivary glands, viral titers increase in the saliva, and eventually reach plateau levels.^27^ Female *Culex* mosquitoes have two salivary glands that each consist of one median and two lateral lobes.^41^ The salivary gland lobes are surrounded by the salivary gland basal lamina, which forms a physical barrier to virus infection of the salivary gland epithelial cells.^35,39,41^ Although less profound than the midgut barrier, the presence of a salivary gland entry barrier has been reported for several flaviviruses in both *Aedes* and *Culex* mosquitoes.^42^^–44^ Both the mechanical and the molecular nature of the salivary gland infection and escape barriers have not been defined completely. Presumably, the combination of basal lamina and organization of the tissue surrounding the salivary glands can prevent viruses from infecting the salivary glands, while antiviral immune pathways can restrict virus replication intracellularly.^35, 45^ In addition, some studies have shown that the release of transmissible virus into the salivary gland lumen might require the induction of apoptosis.^39, 46^

## EUROPEAN VECTOR COMPETENCE STUDIES ON WNV

### Methodology of European vector competence studies for WNV

From the >60 mosquito species that have been implicated as potential WNV vector species in the United States of America (USA),^47^ seven species that also occur in Europe have been experimentally tested for WNV susceptibility (Table 1). From Table 1 it becomes clear that there is large variation in the methodology of different vector competence studies. Vector competence studies have been conducted with mosquitoes collected in the field, or mosquitoes that were reared for many generations in the laboratory. Laboratory colonization can affect vector competence of mosquitoes for viruses.^58, 59^ Laboratory colonies, which generate high numbers of mosquitoes that readily feed via artificial membrane feeders, can provide a useful model system to test for underlying mechanisms of vector competence. However, one should be careful in the extrapolation of results obtained with laboratory colonies to the situation in the field. Thus, use of field-derived mosquitoes is necessary to reliably estimate vector competence of mosquito populations in the field.

Another factor that affects the vector competence is the method of virus stock production, such as the passage number, cell type, and multiplicity of infection. These parameters can affect the composition of the virus population and either reduce or increase virus fitness.^60^ While most studies grow WNV on C6/36 mosquito cells, others generate viral stocks on mammalian Vero cells. Cell lines lack the viral bottlenecks that are present in the vector and host, and therefore affect the quasi-species composition of the viral stock. Moreover, extensive virus passage on a specific cell line may accumulate mutations that have an advantage for viral replication in that specific cell line. Consequently, viral passage on cells can lead to both an increase or decrease in viral infectiousness to mosquito hosts, depending on the type of cells used. For instance, WNV grown on mammalian cells loses fitness in mosquito cells.^61^ It is therefore important that WNV stocks used for vector competence studies have a low number of passages on cells.

Various methods of blood feeding, such as the use of artificial membrane feeders or cotton sticks, have proven to be successful for infection of mosquitoes (Table 1). Such oral exposure techniques are necessary to obtain a reliable proxy for vector competence, because mosquitoes are exposed to WNV via an infectious blood meal. Comparison of blood-feeding methods for other virus–vector combinations has indicated that there is an effect on the infection rate depending on the feeding method used.^62^ However, the effect of the feeding technique on the infection rate of mosquitoes for WNV remains to be investigated. Nevertheless, it is important to use a standardized infection protocol that resembles the natural situation. As most laboratories use membrane feeding as a standard technique, we propose to set membrane-based feeding as the standard method for oral infection of *Culex* mosquitoes with WNV. More artificial infection techniques, such as microinjection, are not suitable for the assessment of vector competence, because they circumvent the midgut barrier. However, comparisons between oral exposure and injection techniques can provide important insights in the role of mosquito barriers in WNV infection, dissemination and transmission.

A large diversity of (un)natural blood sources (for example, rabbit blood or washed erythrocytes) is being used to expose mosquitoes to WNV. It has been shown for several arbovirus-vector combinations that the blood source used in an artificial blood meal can affect the vector competence of mosquitoes.^63^ Under natural conditions, transmission of WNV occurs via the bite of a mosquito on an infectious bird. Thus, ideally an avian blood source, such as chicken blood, should be used for studies with WNV. Only four out of the twelve assessed vector competence studies used an avian (chicken) blood source to infect mosquitoes with WNV (Table 1). As the blood source can have consequences for the initial stages of virus infectivity in the midgut cells, it is important to standardize the blood source for vector competence studies. As chicken blood is widely available and most comparable to the blood of natural bird hosts, we propose the use of chicken blood as a standard blood source for vector competence studies with WNV.

Another parameter that affects the outcome of vector competence studies is the viral dose in the infectious blood meal. Generally, a higher infectious dose results in a higher infection rate, and in some cases also a higher transmission rate.^18, 19^ In order to compare the outcomes of different vector competence studies, it is important to standardize the infectious dose of the blood meal. The maximal viral dose of WNV in viraemic birds is highly species-specific and can reach peak titers up to 1.0 × 10^12^ TCID_50_/mL.^64^ However, many bird species will not reach peak viraemia higher than 1.0 × 10^8^ TCID_50_/mL.^64, 65^ As a proxy for natural bird vireamia, we propose to include an infectious dose of 1.0 × 10^7^ TCID_50_/mL in all future vector competence studies of *Culex* mosquitoes for WNV.

Quantification of virus is routinely done by determining the number of viral genomic RNA copies with quantitative reverse-transcription (qRT)-PCR or through determining the amount of infectious virus as either the 50 % tissue culture infectious dose (TCID_50_) or numbers of plaque forming units (PFU). Although there is a correlation between the number of viral RNA copies and infectious virus particles, the viral genome copies can be 100–5000 times higher than the number of truly infectious virus particles.^66, 67^ This can be due to the presence of defective-interfering particles, that can be detected by qRT-PCR but not with an infectivity assay as they are non-infectious. It is therefore highly important to determine the number of infectious virus particles, as only these virus particles play a role in infection. This can be done by either the use of cell infectivity assays or proper validation of qRT-PCR results by means of a standard curve against the number of infectious particles per Ct value.

Most of the vector competence studies report a combination of infection, dissemination, and transmission rates which provide insight in the different stages of viral dissemination linked to the two main barriers inside the mosquito: the midgut and the salivary glands. Infection rates are determined based on the entire mosquito body, and represent successful infection of the midgut cells. Dissemination rates can be determined by testing peripheral mosquito body parts, such as the head or legs, and provides insight in the proportion of mosquitoes in which WNV managed to escape from the midgut cells and disseminate to the tested body part. Transmission rates are determined by testing saliva for the presence of virus, which provides insight in the proportion of mosquitoes that can actually transmit WNV. This is the most relevant parameter for vector competence, as only mosquitoes with infectious saliva will contribute to WNV transmission.

### Vector competence studies on WNV with European mosquito species

WNV transmission rates have only been determined for five European mosquito species: *Ae. albopictus*, *Ae. caspius*, *Ae. detritus*, *Cx. modestus* and *Cx. pipiens* (Table 2). Seven out of nine studies reporting WNV transmission rates were conducted with the species *Cx. pipiens*, which has been identified as one of the most important WNV vectors in the USA and Europe.^68,69^ In this review, we will focus on results of those studies reporting transmission rates, because only transmission rates can provide a reliable proxy for the capability of a mosquito to transmit WNV. Overall, WNV transmission rates of European *Cx. pipiens* mosquitoes ranged between 0 and 60 % (Table 2).^22^^,49, 53, 54, 55, 56^ Maximum transmission rates of northern European populations were 33 %,^54, 56^ compared to up to 60 % for southern European populations.^49^ Part of this variation may be explained by intrinsic differences in vector competence of geographically distinct populations, or may be due to differences in study protocols (for example, viral strain, virus dose, incubation time or incubation temperature). Distinct populations of mosquitoes may differ in the effectiveness of their midgut and salivary gland barriers or may impose a stronger antiviral immune response, resulting in variation in vector competence. However, in a systematic attempt to directly compare transmission rates of a northern and southern European population, thereby excluding all variation due to experimental design, no actual difference was observed in the transmission rates of both populations.^57^ This suggests that there is no intrinsic difference in vector competence between northern and southern European *Cx. pipiens* populations. However, follow-up studies are needed to verify these results on a broader European scale.

Temperature is another important factor that can influence vector competence. The positive effect of temperature on vector competence has been well established by previous American studies on WNV, and other arboviruses (reviewed in Kenney and Brault^17^). Several studies confirmed that vector competence of European mosquitoes for WNV increases with temperature.^54, 55, 56^ In the temperature range from 18 °C to 28 °C, transmission rates of northern European *Cx. pipiens* increased from 0 % to 33 %.^54, 56^ Thus, average northern European summer temperatures of 18 °C appear to be an important limiting factor for WNV transmission.^54, 55, 56, 57^

The species *Cx. pipiens* comprises two behaviorally different biotypes, *pipiens* and *molestus*, which can form hybrids. The *pipiens* biotype has a preference for birds as hosts,^70, 71^ and is thus important for the natural WNV transmission cycle. Biotype *molestus* and hybrids are thought to play a more important role in the spill over of WNV from birds to humans, due to the preference of the *molestus* biotype for mammals and the more opportunistic feeding behaviour of hybrids.^70, 71^ Thus far, two studies reported transmission rates of *Cx*. *pipiens* mosquitoes which were identified to the biotype level.^56, 57^ One of these studies revealed that overall there is no difference in vector competence among the two biotypes in the Netherlands.^56^ However, the two biotypes did respond differentially to temperature. Higher temperatures increased the transmission rates of biotype *pipiens* and hybrids, but not of biotype *molestus*.^56^ This shows the importance of identifying *Cx. pipiens* to the biotype level and suggests that closely related mosquitoes may have different vector competence. Thus, the *Cx. pipiens* biotypes provide an interesting model system for comparative studies on the underlying genetic and molecular mechanisms of temperature-dependent vector competence.

Transmission rates have also been determined for *Cx. modestus*, originating from France. Relatively high transmission rates of 40–55 % were found, which indicates that *Cx. modestus* is an efficient vector of WNV.^22, 23^ However, *Cx. modestus* was exposed to high WNV titers (10^10^ PFU/mL) in both studies, which can explain the relatively high transmission rates compared to other mosquito species which were exposed to lower WNV titers.

Vector competence for WNV has only been determined for a few European *Aedes* species. An Italian population of the invasive mosquito species *Ae. albopictus* shows variation in transmission rates ranging between 0 % and 40 %, which can be explained by different incubation periods.^49^ Although *Ae. albopictus* mosquitoes are competent vectors for WNV, their contribution to WNV transmission in the field is expected to be low due to their strong feeding-preference for human hosts.^11^ An *Ae. detritus* population from the United Kingdom had moderate transmission rates of 21 %.^50^ Dissemination and transmission rates of *Ae. caspius* were 1 % and 0 %,^22^ respectively, and infection rates of *Ae. japonicus japonicus* were 0 %,^51^ which indicates that these European populations are not competent. This contrasts to the relatively high transmission rates found for *Ae. j. japonicus* in the USA.^72, 73^ To summarize, *Aedes* mosquitoes do not seem to contribute significantly to WNV circulation in Europe.

## UNDERLYING MECHANISMS OF VECTOR COMPETENCE OF *CULEX* MOSQUITOES FOR FLAVIVIRUSES

Vector competence of a given mosquito vector depends on the ability of the virus to overcome the mosquito barriers to transmission. It is therefore important to understand the nature of these barriers and how they interfere with virus dissemination. One way to study the role of the midgut barrier is to compare infection rates between orally exposed and intrathoracically injected mosquitoes. Several studies have shown that intrathoracic injections of WNV into northern European *Cx. pipiens* mosquitoes results in infection rates of ~100 %, as compared to up to 63 % after an infectious blood meal where the virus has to escape from the midgut lumen by infecting midgut epithelial cells.^54, 55, 56, 74^ This indicates that *Cx. pipiens* mosquitoes have a midgut infection barrier for WNV.

The same studies show that WNV transmission rates (WNV presence in saliva) of European *Cx. pipiens* are lower compared to WNV infection rates (WNV presence in body) after oral exposure. This suggests the presence of either a midgut escape barrier, a salivary gland barrier, or both. The presence of a salivary gland barrier can be assessed by comparing transmission rates between orally exposed and injected mosquitoes. In the absence of a salivary gland barrier, mosquitoes intrathoracically injected with WNV would have transmission rates of ~100 %. Indeed, it has been repeatedly shown that intrathoracic injections result in transmission rates of ~100 % in *Cx. pipiens* mosquitoes, indicating the absence of a salivary gland barrier for WNV transmission.^54, 55, 56, 74^ However, studies on *Cx. modestus* suggest the presence of a salivary gland barrier, as they reach only 40–50 % transmission rates whereas dissemination rates were 89–91 %.^22, 23^ As the vector competence of European mosquitoes is dependent on the ability of the virus to overcome the midgut and salivary gland barriers, it is important to understand the underlying mechanism of these barriers.

### Innate immune responses

As a defence to incoming pathogens, mosquitoes have evolved several antiviral responses, such as the RNA interference (RNAi), Janus Kinase/Signal Transducer and Activator of Transcription (JAK/STAT), Toll, immune deficiency (IMD) and apoptosis pathways.^75^ In addition, experimental evidence suggests that heat-shock proteins and reactive oxygen species also have antiviral properties (reviewed in Saraiva *et al.*^76^), but due to the limited knowledge available on these responses they will not be discussed here. The expression of genes that encode proteins and factors that function in the mosquito innate immune pathways can be upregulated or activated after ingestion of an infectious blood meal. In this way, these immune pathways play an important role in the midgut and possibly the salivary gland barriers that in turn contribute to the vector competence of a mosquito for certain arboviruses. Different mosquito species can have variation in the effectiveness of their antiviral immune responses against certain arboviruses. Such variation might explain differences in vector competence of different mosquito species for certain viral strains. Most knowledge on the invertebrate immunity is derived from research on *Drosophila,* although there is an increasing number of studies on immunity against alphaviruses and flaviviruses, mostly conducted on *Aedes* mosquitoes. Here we review the literature concerning flavivirus infections and their role in triggering the mosquito antiviral immune responses in both *Aedes* and *Culex* species mosquitoes.

RNAi is one of the most important immune responses of invertebrate vectors.^77^ The RNAi response is an antiviral response of insects that is induced upon recognition of non-self double-stranded (ds)RNA. Even though flaviviruses are single stranded (ss)RNA viruses, dsRNA intermediates are produced during viral RNA replication that serve as substrates for the endoribonuclease Dicer-2 to activate the RNAi response. In addition, the genomic RNA can fold into dsRNA secondary structures that can be recognized by the RNAi machinery of the mosquito. Activation of the RNAi response leads to degradation of viral genomic RNA through cleavage by Argonaute-2, resulting in decreased virus replication. Various studies have shown that viral small-interfering RNAs are produced during infection of Usutu virus, DENV-2, and WNV in mosquitoes.^54, 74, 78^ This indicates that the RNAi response is initiated upon infection of arboviruses in mosquitoes, and can influence the susceptibility of these mosquitoes for arbovirus infection. RNAi is capable of controlling DENV infection in *Ae. aegypti* mosquitoes and results in decreased viral titers and transmission rates.^79^ In addition, silencing of important effector proteins in the RNAi pathway increases DENV-2 infection rates and titers in *Ae. aegypti*.^80^ Interestingly, in *Cx. quinquefasciatus* RNAi targets the WNV genomic RNA in the mosquito midguts.^81^

As RNAi is a potent antiviral response that restricts flavivirus transmission, it is not surprising that several RNAi suppressors have been identified in flavivirus genomes. For example, yellow fever virus capsid proteins can bind to double-stranded RNA and thereby could suppress RNA silencing, while for DENV the NS4B protein was identified as an RNAi suppressor.^82, 83^ WNV encodes for an RNAi suppressor in the form of a highly structured subgenomic RNA, that acts as a decoy substrate for Dicer and thereby prevents induction of the RNAi response.^74^ Correspondingly, a mutant WNV that lacks a potential viral suppressor of RNAi (based on *in vitro* studies) has decreased transmission rates after an infectious blood meal, but not after intrathoracic injection.^74^ This observation suggests that WNV requires this potential RNAi suppressor to suppress the RNAi response in the mosquito midgut and thereby overcome the midgut barrier.

The invertebrate JAK/STAT pathway is the insect innate immune pathway that resembles the mammalian interferon response.^84^ The JAK/STAT pathway is initiated upon virus recognition by a transmembrane receptor that activates a signaling cascade, which upregulates the expression of antiviral response genes. These antiviral genes act by recognizing virus infection, suppressing virus replication, and by triggering nearby cells to enter an antiviral state. Although strongly established as an antiviral defense system in *Drosophila* (reviewed in Arbouzova *et al*^85^), the influence of the JAK/STAT pathway on flavivirus infection in mosquitoes remains to be resolved. One study with DENV-2 in *Ae. aegypti* mosquitoes showed that silencing either components or effectors of the JAK/STAT pathway increases DENV-2 titers, while silencing of a JAK/STAT antagonist decreases DENV-2 titers.^86^ This suggests that under natural conditions, the *Ae. aegypti* JAK/STAT pathway restricts DENV-2 and influences the vector competence. In *Ae. aegypti*, infection with WNV and DENV has been shown to modulate the expression of JAK/STAT pathway-related genes.^87, 88^ WNV infection in *Cx. quinquefasciatus* activates the JAK/STAT transcription factor STAT1A, suggesting that the JAK/STAT pathway is involved in controlling WNV infection. However, this activation has not been shown to restrict WNV dissemination or transmission.^89^ In *Cx. quinquefasciatus,* activation of the RNAi pathway increases expression of the cytokine Vago, which is secreted from infected cells, to subsequently activate the JAK/STAT signaling cascade in neighbouring cells. Through this indirect mechanism, Vago thus decreases WNV titers in a JAK/STAT-dependent manner,^90^ supporting the role of the JAK/STAT pathway in controlling flavivirus infections in *Culex* mosquitoes.

Another conserved immune response in mosquitoes is the Toll pathway. The Toll pathway is activated after recognition of virus infection by Toll-like receptors. This eventually results in the expression of antiviral effector genes (reviewed in Sim *et al.*^91^). DENV-2 infection of *Ae. aegypti* mosquitoes activates the main components of the Toll pathway by increasing their transcriptional level.^88^ Depletion of Toll regulators results in increased DENV-2 and DENV-4 titers, whereas silencing of a Toll pathway inhibitor results in decreased DENV titers.^88, 92^ This indicates that the Toll pathway restricts flavivirus replication in mosquitoes and could influence the vector competence of mosquitoes. DENV infection of salivary glands in *Ae. aegypti* results in upregulation of Toll pathway-related genes in the salivary glands, indicating that the salivary glands can mount an antiviral response against flaviviruses.^45^ Transcriptomic approaches have shown that components of the Toll pathway are also differentially expressed during yellow fever virus, but not WNV infection.^87^ In another study, infection of *Cx. quinquefasciatus* with WNV alters the regulation of various genes, but Späetzle-like genes were the only modulated Toll pathway-related genes.^89^ Future studies should identify whether the Toll pathway truly restricts flavivirus transmission by mosquitoes and whether the Toll pathway is also involved in restriction of WNV by *Culex* mosquitoes.

The IMD pathway is activated upon recognition of virus infection by the adaptor IMD protein, subsequently resulting in the transcription of IMD effector genes (reviewed in Sim *et al.*^91^). Studies with DENV-2 have shown that infected *Ae. aegypti* have increased expression of IMD pathway components.^88, 93^ However, silencing of the IMD antagonist Caspar does not affect DENV-2 titers, suggesting that the IMD pathway is not involved in controlling DENV-2 infection in *Ae. aegypti.*^88^ Interestingly, silencing of the IMD pathway in a DENV refractory *Ae. aegypti* population resulted in increased DENV-2 replication suggesting that the IMD pathway contributes to the refractoriness of this mosquito population.^94^ WNV infection in *Cx.quinquefasciatus* does not affect the regulation of IMD related genes,^89^ although more studies are required to understand the role of the IMD pathway during WNV infections in *Culex.*

Apoptosis (programmed cell death) is considered an antiviral response by the host to eliminate viral infection by killing infected and neighboring cells. Many viruses inhibit apoptosis in order to facilitate their replication and to prevent clearance of the infected cells.^46^ Interestingly, some arboviruses utilize cellular caspases during replication or use apoptosis as a measure to release virions from infected cells. WNV infection in *Cx. pipiens* causes apoptosis in the midgut cells, mainly in the anterior region.^95^ Infection of *Ae. aegypti* mosquitoes with DENV results in upregulation of apoptosis-related genes. For some genes that function in the apoptosis pathway, silencing results in increased DENV replication.^96^ In addition, DENV infection of both a refractory and susceptible strain of *Ae. aegypti* induces the expression of the apoptotic gene *mx* as soon as 3 h post blood meal in the refractory, but not in the susceptible strain.^97^ These observations suggest that apoptosis acts as an antiviral response in the midgut. Also, in the salivary glands, WNV infection causes apoptotic-like cell death, most likely due to the triggering of cellular apoptotic pathways as a result of active virus replication.^28, 98^ In addition, infection of *Cx. quinquefasciatus* salivary glands with WNV results in a decrease in a *Culex* apoptosis inhibitor.^99^ Although apoptosis is, thus, induced in the salivary glands, virus titers do not rapidly decline over time, suggesting that apoptosis is not effectively clearing the virus-infected cells in the salivary glands.^98^ Alternatively, virus-induced apoptosis in the salivary glands can perhaps increase the release of transmissible virus into the saliva thereby actually facilitating virus transmission. Future studies should elucidate the effect of apoptosis on WNV infection of the midgut and release of WNV into the saliva.

### The mosquito microbiome

Mosquitoes live in symbiosis with a wide variety of bacteria and fungi, which are abundantly present in the mosquito gut and are referred to as the mosquito gut microbiome.^100^ These microorganisms interact with the mosquito gut and have a strong impact on the mosquito’s metabolism, but also affect interactions with pathogens.^101^ The microbiome of *Ae. aegypti* is important for successful infection of flaviviruses, as has been shown for DENV-2.^100^ The introduction of specific bacterial strains into the mosquito gut microbiome can affect the infection or replication of flaviviruses in mosquitoes either in a positive or negative way, which in turn may affect their vector competence for certain viruses.^100^ In addition, mosquitoes can be persistently infected with insect-specific viruses that normally do not affect the mosquito life cycle, but can have an effect on the ability of the mosquito to become infected with other viruses.^102^

Apart from bacteria in the mosquito midgut, some bacteria are present inside the cells of mosquitoes and are transmitted both horizontally between species and vertically to the mosquito offspring.^103^ The best documented intracellular bacterium of mosquitoes is *Wolbachia*. *Wolbachia* is an endosymbiotic bacteria that can affect vector competence for arboviruses (reviewed in Johnson^104^). Although there is a large body of literature describing the interactions between flaviviruses and *Wolbachia*, we will here discuss those studies addressing the effect of *Wolbachia* on transmission and replication of WNV only. *Wolbachia* infection increased WNV replication, but resulted in >100-fold less infectious virus production, suggesting that *Wolbachia* interferes with virion assembly within or secretion of virus from mosquito cells.^105^ Consequently, *Ae. aegypti* mosquitoes infected with *Wolbachia* have ~50 % reduction in WNV infection and 100 % reduction in transmission rates. Infection of *Cx. quinquefasciatus* with *Wolbachia* results in increased resistance to WNV infection via a blood meal. Dissemination rates and transmission rates were significantly lower (2–3-fold) as compared to mosquitoes cleared from *Wolbachia* with tetracycline.^106^ However, *Cx. tarsalis* mosquitoes infected with *Wolbachia* showed 1.5–2-fold enhanced WNV infection rates due to downregulation of Toll pathway-related genes by *Wolbachia*.^107^ Accordingly, a study on *Wolbachia* in field-caught *Cx. quinquefasciatus* populations showed that densities (that is, the bacterial load) of *Wolbachia* were too low to inhibit WNV infection and did not affect vector competence of these same mosquito colonies. Thus, for *Cx. quinquefasciatus* the virus inhibitory effect depends on the density of *Wolbachia* in the mosquito.^108^ The effect of *Wolbachia* on vector competence of mosquitoes is further dependent on the strain of *Wolbachia* in combination with the species of mosquito used.^104^ Taken together infection with *Wolbachia* can have a strong effect on vector competence of mosquitoes for WNV, although the mechanism remains unclear.

## DISCUSSION AND CONCLUSIONS

Until now, four European mosquito species, *Ae. albopictus*,^49^ *Ae. detritus*,^50^ *Cx. modestus*^22, 23^ and *Cx. pipiens*,^49, 53, 54, 55, 56, 57^ have been confirmed to be competent to transmit WNV. In contrast, *Ae. caspius* and *Ae. japonicus japonicus* are not competent as vector for WNV.^22, 51^ Highest transmission rates were found for *Cx. pipiens*, which also have high WNV infection rates in the field,^109^ and are highly abundant during summer.^110^ Therefore, *Cx. pipiens* is considered to be the most important vector for WNV in Europe.

No intrinsic differences in vector competence were found in a direct comparison between a northern and southern European *Cx. pipiens* population.^57^ Importantly, increased WNV transmission was observed at higher temperatures for both northern and southern European *Cx. pipiens.*^57^ This confirms that temperature is likely the most important factor to explain why WNV outbreaks have thus far been limited to southern and central Europe.^55^ Considering the presence of intrinsically competent mosquito vectors,^22, 23, 49, 50, 53, 54, 55^ susceptible bird hosts^6, 7^ and predictions of more frequent and prolonged temperature anomalies, there are no limiting factors for future WNV circulation in northern Europe. In fact, the recent circulation of Usutu virus in northern Europe during the autumn of 2016,^111^ exemplifies that there are no evident restrictions for *Culex*-borne flaviviruses to become endemic in northern Europe. Because of the similar transmission cycle and comparable transmission rates of both Usutu and West Nile virus with their main vector *Cx. pipiens*, the circulation of Usutu virus can be considered as a prelude to WNV transmission.^54^

### Implications for WNV surveillance

Insights in vector competence allow for targeted surveillance and control of highly competent mosquito species which pose a high risk for WNV transmission. Several European countries such as Italy and Greece, have implemented WNV surveillance programmes by monitoring mosquitoes, (sentinel-)birds, (sentinel-)equines, or humans for presence of virus, seroconversion, or disease symptoms. With the knowledge presented in this review, we recommend to implement WNV surveillance programmes throughout Europe. Examples of such surveillance programmes for both northern (for example, United Kingdom) and southern European countries (for example, Italy and Greece) were reviewed in (Engler *et al.*^109^ and Gossner *et al.*^112^). We favour integrating national surveillance initiatives into a Europe-wide surveillance network, as reviewed in (Engler *et al.*^109^ and Gossner *et al.*^112^). This should result in the use of general protocols, which allows for comparisons across Europe. However, selection of appropriate surveillance tools should still be based on the specific needs of individual countries.

Surveillance programmes can either be active through the screening for virus in living organisms or passive, involving the screening of dead birds or based on serology.^109^ Such screening techniques would rely on the detection of WNV by PCR, or detection of WNV-specific antibodies (serology) in humans, equines and birds. Active screening of mosquitoes or sentinel-birds allows for detection early in the season, whereas passive screening of dead birds or cases of neurological disease in equines or humans is most useful to provide insight in emergence of WNV into new areas. Detection early in the season allows for awareness, targeted mosquito control and personal protection which can reduce WNV outbreaks in humans later in the season. This form of surveillance is, therefore, highly recommended for countries, which encounter yearly cases of WNV in humans. However, it is less essential for countries in which no human cases have yet emerged. Active mosquito surveillance is costly and WNV is usually only detected in a few percent of mosquito pools,^109^ even during outbreaks. This makes passive surveillance of suspected cases more suitable for countries without known cases of WNV infection in humans.

### Recommendations for vector competence studies

Considering the vector competence studies of European mosquito species for WNV, it becomes clear that the large variation in study protocols makes it difficult to directly compare studies. Variation in techniques, and choice of mosquito populations and viral strains, have an effect on the outcomes of vector competence studies. To enable comparison between vector competence studies, detailed information on the mosquito vector, virus strain and applied techniques need to be reported. Such information should at the minimum include accurate identification of the mosquito vector, exact origin of mosquito populations (coordinates), mosquito generation, mosquito rearing protocols, incubation temperature and incubation time. Information regarding the virus isolate should include the viral strain used (including GenBank reference), virus passage number, cell types used to grow virus stocks and technical details on how the virus stock was generated. Technical details should include detailed descriptions of blood feeding methods (including the blood source) and infectivity assays. Studies should at the minimum report summary statistics that include number of replicates, sample sizes, mean or median and variation in data. In addition, raw data should be made publicly available, to allow for quantitative meta-analyses. Future research consortia should discuss and adjust research protocols in order to increase comparability between studies from different laboratories. Inclusion of more detailed information and consistency in research protocols allows for better comparison between studies and makes it easier to draw conclusions on observed results.

Choice of incubation time, incubation temperature, and virus dose can strongly influence the outcome of vector competence studies. Studies that focused on the effect of incubation time,^49^ incubation temperature^54, 55, 56, 57^ and WNV dose confirmed their effects on transmission rates.^113^ For studies aiming to determine vector competence of a certain mosquito species, we recommend to use fixed incubation periods and temperatures in order to increase comparability between studies. On the basis of previous studies, we suggest a standardised incubation period of 14 days, which was shown to be sufficiently long for temperatures of 18 °C and higher.^114^ Furthermore, we suggest a standardised incubation period of 27±1 °C. Additional incubation temperatures that represent average summer temperatures of the region the mosquito population originates from are also advised.Importantly, when assessing relatively low incubation temperatures of below 18 °C, the minimum incubation period needs to be increased. Finally, we suggest a standardised WNV dose of 10^7^ TCID_50_/mL. Standardizing vector competence protocols will largely increase the ability to make comparisons between studies, which is essential to increase the understanding on WNV transmission in Europe.

Vector competence studies can provide information such as infection, dissemination and transmission rates, which provides insights in the likelihood of the vector to transmit the virus in the field. Some studies also include the rate at which a model organism can become infected by a viraemic mosquito, which gives a better proxy for transmission in the field. However, these studies are labour intensive, and can often only be performed with a small number of individuals due to ethical constraints and costs. Therefore, assessing the presence of virus in the saliva is the best alternative to estimate transmission rates. In addition, vector competence studies should focus on using cell culture to assess infectivity instead of relying on qRT-PCR methods. Finally, we strongly recommend that future vector competence studies determine transmission rates by measuring virus in the saliva in order to better understand the state of WNV transmission in Europe.

### Future directions for understanding vector competence

In this review, we have shown that European mosquito species vary in their level of competence for WNV, and that some species are not competent. Although this indicates that surveillance for WNV in European mosquitoes is necessary, it does not answer the question why some European vectors are more competent than others. In order to fully understand arbovirus transmission dynamics, it is important to identify the determining factors of vector competence. Especially the influence of the mosquito barriers, immune responses (specifically against flaviviruses), and microbes on vector competence are still poorly understood. Studying the underlying mechanisms of how these barriers and responses influence vector competence in mosquitoes is therefore important to understand variation in vector competence.

As the most important vector species for WNV transmission in Europe is *Cx. pipiens,* more research on virus-vector interactions in *Cx. pipiens* is required to overcome the challenges related to virus control. Fortunately, the genome sequence of *Cx. quinquefasciatus*, the first sequenced genome of a *Culex* species, is available and allows for genetic studies with *Culex* mosquitoes. However, as the genome sequence of *Cx. pipiens* is not available, genetic studies should be performed with *Cx. quinquefasciatus* mosquitoes. Therefore, sequencing of the *Cx. pipiens* genome is an important research goal. Alternatively, attempts can be made to perform *de novo* transcriptomics in *Cx. pipiens* and compare the outcomes with the annotation of the *Cx. quinquefasciatus* genome, although such an approach is likely to miss some candidate genes due to genetic variations.

Thus far, most studies on mosquito immune pathways involved in flavivirus infections have been done with *Aedes* mosquitoes. The limited research on mosquito immune pathways in *Culex* has confirmed the presence of the RNAi, JAK/STAT, Toll, and IMD pathway, as putative antiviral responses towards flavivirus infections. Yet, the relative contribution and effectiveness of these pathways in controlling transmission of WNV and other flaviviruses by *Culex* mosquitoes is still unclear and requires further investigation. Potentially, such studies can indicate novel antiviral responses that are unique for *Culex* species such as the antiviral *Vago* response that has thus far only been observed in *Culex* mosquitoes.^90^ Studies that aim to understand the molecular interactions between the vector and the virus often rely on controlled experiments in cell lines. *Culex* cell lines are available for the species *Cx. quinquefasciatus* (Hsu) and *Cx. tarsalis*(CxT), but a cell line of *Cx. pipiens* is not available. This limits studies on immune responses and virus replication in *Cx. pipiens* mosquitoes to extrapolations from cell lines of other *Culex* species. Because *Cx. pipiens* is the predominant vector species for WNV in Europe, a *Cx. pipiens* cell line would greatly contribute to research that aims to understand the basis of vector competence for WNV in European mosquitoes.

Studies that investigated the effect of temperature on the transmission of WNV by European mosquitoes have shown that increasing temperatures result in higher transmission rates (Table 2). Indeed, higher temperatures can lead to higher virus replication rates. Alternatively, temperature can have an effect on immune regulatory pathways or the microbiome, resulting in altered vector competence.^115^ Interestingly, it has been shown that at low rearing temperatures of 18 °C, the RNAi pathway is less effective in *Ae. aegypti* resulting in increased susceptibility to arbovirus infection.^116^ Similar studies in *Culex* mosquitoes could highlight why the vector competence changes with alternating temperature or whether it is simply the lowered virus replication at lower temperatures. Apart from temperature also the viraemic dose and genetic differences between mosquito populations might affect the induction of innate immune responses. Although it seems natural that a higher viral dose in the initial infection would lead to a stronger induction of immune responses, research supporting this hypothesis is currently lacking. Furthermore, mosquito populations that are more susceptible to WNV infection could have a lower induction of antiviral immune responses upon viral infection. Future studies should try to answer these questions in order to increase our understanding of WNV transmission by mosquitoes. Finally, it has been shown that the composition of the larval environment such as availability of nutrition, larval density, and temperature during development can influence the vector competence of mosquitoes.^117^ The effect of nutrition appeared to be negligible, while competition in the larval environment increases the vector competence. Interestingly, warm rearing temperatures decrease the susceptibility of mosquitoes for infection, in contrary to the influence of temperature during the extrinsic incubation period. Whether differences in larval environment also influence innate immune responses, and thereby alter the vector competence in mosquitoes requires further research.

Understanding the molecular mechanisms of virus transmission allows for more directed control approaches by for example, targeting barrier specific genes or application of microorganisms that reduce vector competence. Various vector control strategies have been proposed based on our current understanding of the mosquito’s immune system such as (i) the development of transmission blocking vaccines, (ii) the generation of refractory mosquitoes through genetic modification of either the mosquito or (iii) the mosquito’s microbiome and (iv) infection of mosquitoes with intracellular bacteria such as *Wolbachia*.^104, 118, 119^ Although no attempts have been made to employ the mosquito’s immune system for the control of arboviral disease, promising results have been made in field studies with *Wolbachia*-infected mosquitoes. Studies with *Wolbachia*-infected *Aedes* mosquitoes in Australia and Vietnam have already shown the potential of *Wolbachia* to invade the local mosquito population.^120^ While the effect on virus-transmission in the field is yet to be determined, this study indicates the feasibility of releasing *Wolbachia*-infected mosquitoes as an arboviral control strategy. Potentially, similar release strategies could be performed with genetically modified mosquitoes that employ the mosquito’s immune system to further prevent the transmission of arboviruses.

## Figures and Tables

**Table 1:** Overview of vector competence studies on West Nile virus with European mosquito species

**Mosquito species**	**Mosquito origin (filial number)**	**WNV lineage (passage number)**	**Cell type virus propagation**	**Feeding method**	**Blood type**	**Infection assessment**	**Reported rates**	**References**
*Aedes albopictus*	Spain (>F30)	Lineage 1 and 2 (−)	C6/36 or Vero E6	Hemotek with chicken skin membrane	Heparinised bovine blood with ATP	qRT-PCR	IR and DR	^48^
	Italy (−)	Lineage 1 (−)	-	Glass feeder with pig-intestine membrane	-	qRT-PCR	IR, DR, and TR	^49^
*Aedes caspius*	France (F0)	Lineage 1 (P5)	C6/36	Chicken skin membrane	Washed rabbit erythrocytes with ATP	IFA or qRT-PCR	DR and TR	^22^
*Aedes detritus*	United Kingdom (F0)	Lineage 1 (−)	Vero E6	Odorised feeding membrane	Heparinised human blood	qRT-PCR	TR	^50^
*Aedes japonicus japonicus*	Germany (F0)	Lineage 1 (−)	-	Cotton stick	Human blood with fructose and fetal calf serum	qRT-PCR	IR	^51^
*Culex modestus*	France (F3–4)	Lineage 1 (P5)	C6/36	Chicken skin membrane	Washed rabbit erythrocytes with ATP	IFA or qRT-PCR	DR and TR	^22, 23^
*Culex torrentium*	Germany (F0)	Lineage 1 (−)	-	Cotton stick	Human erythrocytes with fructose and fetal calf serum	qRT-PCR	IR and DR	^52^
*Culex pipiens s.l.*	France (F1)	Lineage 1 (P5)	C6/36	Chicken skin membrane	Washed rabbit erythrocytes with ATP	IFA or qRT-PCR	DR and TR	^22^
	Italy (F0, F7, or F11)	Lineage 1 (−)	Vero E6	Glass feeder with pig-intestine membrane	Rabbit blood with EDTA	qRT-PCR	IR, DR, and TR	^53^
	Italy (−)	Lineage 1 (−)	-	Glass feeder with pig-intestine membrane	-	qRT-PCR	IR, DR, and TR	^49^
	The Netherlands (−)	Lineage 2 (P2)	C6/36	Hemotek with Parafilm membrane	Chicken blood	CPE	IR and TR	^54^
	The Netherlands (−)	Lineage 1 and 2 (P2)	C6/36	Hemotek with Parafilm membrane	Chicken blood	CPE and IFA	IR and TR	^55^
*Culex pipiens pipiens*	Germany (F0)	Lineage 1 (−)	-	Cotton stick	Human erythrocytes with fructose and fetal calf serum	qRT-PCR	IR and DR	^52^
	The Netherlands (F3-F5)	Lineage 2 (P2)	C6/36	Hemotek with Parafilm membrane	Chicken blood	CPE	IR and TR	^56^
	The Netherlands (F4-F6)	Lineage 2 (P2)	C6/36	Hemotek with Parafilm membrane	Chicken blood	CPE	IR and TR	^57^
	Italy (F4-F6)	Lineage 2 (P2)	C6/36	Hemotek with Parafilm membrane	Chicken blood	CPE	IR and TR	^57^
*Culex pipiens molestus*	Spain (>F30)	Lineage 1 and 2 (−)	C6/36 or Vero E6	Hemotek with chicken skin membrane	Heparinised bovine blood with ATP	qRT-PCR	IR and DR	^48^
	Germany (−)	Lineage 1 (−)	-	Cotton stick	Human erythrocytes with fructose and fetal calf serum	qRT-PCR	IR	^52^
	The Netherlands (−)	Lineage 2 (P2)	C6/36	Hemotek with Parafilm membrane	Chicken blood	CPE	IR and TR	^56^
*Culex pipiens* Hybrid *(pipiens* x *molestus)*	Spain (>F30)	Lineage 1 and 2 (−)	C6/36 or Vero E6	Hemotek with chicken skin membrane	Heparinised bovine blood with ATP	qRT-PCR	IR and DR	^48^
	The Netherlands (F1)	Lineage 2 (P2)	C6/36	Hemotek with Parafilm membrane	Chicken blood	CPE	IR and TR	^56^

Abbreviations: cytopathic effect, CPE; dissemination rate, DR; immunofluorescence assay, IFA; infection rate, IR; quantitative reverse transcription-PCR, qRT-PCR; transmission rate, TR. Hyphen indicates that used variable was not reported in the study.

Listed are important methodological variables that were used in studies with European mosquito species.

**Table 2:** Transmission rates of European mosquito species for West Nile virus

**Mosquito species**	**Mosquito origin**	**WNV lineage (Origin, year)**	**WNV titer in blood meal**	**Incubation temperature**	**Incubation period**	**Infection rate (n)**	**Dissemination rate (n)**	**Transmission rate (n)**	**References**
*Aedes albopictus*	Italy	Lineage 1 (Italy, 2011)	9.3 × 10^7^ PFU/mL	27 °C	0	100 % (5–7)	0 % (5–7)	0 % (5–7)	^49^
					3	20 % (5–7)	0 % (5–7)	0 % (5–7)	
					9	40 % (5–7)	40 % (5–7)	0 % (5–7)	
					14	80 % (5–7)	40 % (5–7)	40 % (5–7)	
					21	20 % (5–7)	20 % (5–7)	20 % (5–7)	
					28	20 % (5–7)	20 % (5–7)	20 % (5–7)	
*Aedes caspius*	France	Lineage 1 (France, 2000)	2.0 × 10^10^ PFU/mL	26 °C	14	-	1 % (124)	0 % (42)	^22^
*Aedes detritus*	United Kingdom	Lineage 1 (New York, 1999)	2.0 × 10^6^ PFU/mL	21 °C	17	-	-	21 % (78)	^50^
*Culex modestus*	France	Lineage 1 (France, 2000)	2.0 × 10^10^ PFU/mL	26 °C	14	-	91 % (22)	40 % (5)	^23^
						-	89 % (37)	55 % (22)	^22^
*Culex pipiens s.l.*	France	Lineage 1 (France, 2000)	2.0 × 10^10^ PFU/mL	26 °C	14	-	39 % (234)	16 % (57)	^22^
	Italy	Lineage 1 (Italy, 2011)	9.3 × 10^7^ PFU/mL	27 °C	0	100 % (5–7)	0 % (5–7)	0 % (5–7)	^49^
					3	100 % (5–7)	0 % (5–7)	0 % (5–7)	
					9	100 % (5–7)	0 % (5–7)	0 % (5–7)	
					14	60 % (5–7)	20 % (5–7)	20 % (5–7)	
					21	80 % (5–7)	80 % (5–7)	60 % (5–7)	
					28	71 % (5–7)	71 % (5–7)	43 % (5–7)	
				28 °C	32	55–90 % (112)	28–90 % (112)	21–33 % (112)	^53^
	The Netherlands	Lineage 2 (Greece, 2010)	4.0 × 10^7^ TCID_50_/mL	28 °C	14	46 % (-)	-	33 % (-)	^54^
		Lineage 1 (New York, 1999)	1.4 × 10^8^ TCID_50_/mL	23 °C	14	49 % (131)	-	22 % (67)	^55^
		Lineage 2 (Greece, 2010)	1.4 × 10^8^ TCID_50_/mL	23 °C	14	33 % (154)	-	24 % (79)	^55^
*Culex pipiens pipiens*	The Netherlands	Lineage 2 (Greece, 2010)	5.7±1.0 × 10^7^ TCID_50_/mL	18 °C	14	29 % (50)	-	0 % (50)	^56^
				23 °C	14	50 % (50)	-	6 % (50)	
				28 °C	14	63 % (50)	-	33 % (50)	
	Italy	Lineage 2 (Greece, 2010)	4.2±1.0 × 10^7^ TCID_50_/mL	18 °C	14	28 % (50)	-	0 % (50)	^57^
				23 °C	14	36 % (50)	-	2 % (50)	
				28 °C	14	32 % (50)	-	16 % (50)	
	The Netherlands	Lineage 2 (Greece, 2010)	4.2±1.0 × 10^7^ TCID_50_/mL	18 °C	14	26 % (50)	-	0 % (50)	^57^
				23 °C	14	34 % (50)	-	10 % (50)	
				28 °C	14	32 % (50)	-	10 % (50)	
*Culex pipiens molestus*	The Netherlands	Lineage 2 (Greece, 2010)	5.7±1.0 × 10^7^ TCID_50_/mL	18 °C	14	24 % (50)	-	6 % (50)	^56^
				23 °C	14	24 % (50)	-	10 % (50)	
				28 °C	14	14 % (50)	-	10 % (50)	
*Culex pipiens* Hybrid (*pipiens* x *molestus*)	The Netherlands	Lineage 2 (Greece, 2010)	5.7±1.0 × 10^7^ TCID_50_/mL	18 °C	14	24 % (50)	-	2 % (50)	^56^
				23 °C	14	39 % (50)	-	0 % (50)	
				28 °C	14	43 % (50)	-	14 % (50)	

Abbreviations: plaque forming units, PFU; tissue culture infectious dose 50, TCID_50_.

Overview of currently available data on vector competence studies which reported transmission rates of European mosquito species, based on assessment of individual mosquitoes. Only data obtained by natural infection (infectious blood meal) are shown. Infection, dissemination, and transmission rates were calculated as the number of positive samples divided by the total number of tested fed female mosquitoes. Hyphen indicates unknown value.
